# Social information as an entrainment cue for the circadian clock

**DOI:** 10.1590/1678-4685-GMB-2024-0008

**Published:** 2024-07-22

**Authors:** Chiara Costa Petrillo, Nicolás Pírez, Esteban J. Beckwith

**Affiliations:** 1 Consejo Nacional de Investigaciones Científicas y Técnicas, Instituto de Fisiología, Biología Molecular y Neurociencias, Buenos Aires, Argentina.; 2 Universidad de Buenos Aires, Facultad de Ciencias Exactas y Naturales, Instituto de Fisiología, Biología Molecular y Neurociencias, Buenos Aires, Argentina.

**Keywords:** Circadian rhythms, entrainment, synchronization, social cues, sensory modalities

## Abstract

Animals adapt to the daily changes in their environmental conditions by means of genetically encoded circadian clocks. These clocks, found throughout the tree of life, regulate diverse biological functions, and allow periodical changes in physiology and behaviour. The molecular underpinnings of these clocks have been extensively studied across taxa, revealing a brain-based system that coordinates rhythmic activities through neuronal networks and signalling pathways. Entrainment, the alignment of internal rhythms with external cues or *zeitgebers*, is crucial for the adaptive value of these internal clocks. While the solar light-dark cycle is a primary *zeitgeber* for most animals, other relevant cues such as temperature, meal timing, predators, anxiety, fear, physical activity, and social interactions also play roles in entraining circadian clocks. The search of a detailed description of the circadian clocks is a goal for neurobiology and an area of growing societal interests. Moreover, as disruptions in circadian rhythms are implicated in various diseases, understanding the entrainment pathways contributes to developing interventions for improved wellbeing and health outcomes. This review focuses on socially relevant cues, examining their impact on animal physiology and behaviour, and explores the sensory pathways transmitting information to the central clock.

## The circadian clock and its entrainment capabilities

Animals exhibit cyclic variations in behaviour and physiology, enabling them to anticipate and adapt to daily changes in their environment. These variations encompass well-known behavioural states like sleep-wake cycles as well as numerous subtle oscillations in neurological, metabolic, endocrine, cardiovascular, and immune functions. The underlying mechanism responsible for generating this behavioural and physiological rhythmicity is a genetically encoded molecular clock. These clocks, present across the tree of life, consist of interconnected components that regulate their own abundance and activity, resulting in rhythms that are close to 24 hours in duration and are hence referred to as circadian (from the Latin *circa* -about- *dies* -a day-) clocks (Kumar, 2017).

Understanding the physiological processes regulating the central circadian clock is of utmost importance. Disruptions in circadian rhythms are associated with numerous diseases that significantly influence both health and the economy. These diseases include sleep disorders, type II diabetes, cardiovascular diseases, autoimmune diseases, depression, bipolar disorders, and many others (Meyer et al., 2022; Rijo-Ferreira and Takahashi, 2019). Therefore, understanding the relationships between the external factors that influence the central clock and their effect on the circadian physiology holds great relevance. In the long term, this will help to elucidate the underlying mechanisms of multiple diseases and will contribute to develop effective interventions to mitigate their impact. 

Extensive research has shed light on the molecular components of the circadian clock across taxa, offering insights into the regulatory machinery behind the generation of the close-to-24-hour rhythms. In animals, the molecular machinery of the circadian clock is primarily located in the brain where specialized cells known as *clock cells* communicate with each other to coordinate their activities and regulate downstream circadian rhythms. The communication within the master pacemaker cells involves intricate networks of neuronal connections and signalling pathways that have different properties depending on the taxa of focus, and have been extensively studied (Cox and Takahashi, 2019; Shafer et al., 2022; Reinhard et al., 2023; Starnes and Jones, 2023). At the molecular level, the cogs of the clock employ a machinery that involves multiple layers of control, ranging from the regulation of epigenomic marks to the oscillation of posttranslational modifications (Beckwith and Yanovsky, 2014). A key common aspect of all circadian systems is that the central neuronal pacemaker synchronizes the rest of the body, influencing multiple tissues and other secondary cell-autonomous oscillators, aiming to align internal rhythms. To achieve a meaningful synchronization, the master circadian clock relies on external stimuli known as *zeitgebers* (German: “time givers”). Thus, *entrainment* is the alignment of the internal biological clock rhythm to external time cues, and key aspects of entrainment are the resulting phase and periodicity of the controlled variables. In lay terms, entrainment is the way that our internal clocks are reset to reflect the natural periods of day and night that occur in our environment. While the daily solar light-dark cycle serves as the primary *zeitgeber* for many animals, the relevance of the other external cues is determined by the physiology and ecology of each species, and it is shaped through evolution. Thus, animals also respond to other cues such as temperature, meal timing, presence of predators, elements that produce anxiety or fear, their own physical activity, and social interactions to entrain their circadian clock (Kumar, 2017).

The focus of this review will be on the relevant cues generated by social interactions that can influence the phase of physiology and behaviour in animals, and what we know about the sensory pathways that convey the information from the periphery to the master clock. Since the knowledge on circadian clock and their entrainment pathways is vast, we will refer the reader to wonderful reviews and textbooks conducting an in-depth study of how other relevant cues entrain the circadian clock (Bloch et al., 2013; Kumar, 2017; Foster et al., 2020).

## Social interactions are key factor influencing fitness, are they relevant for the circadian clock?

**Figure 1 -:**
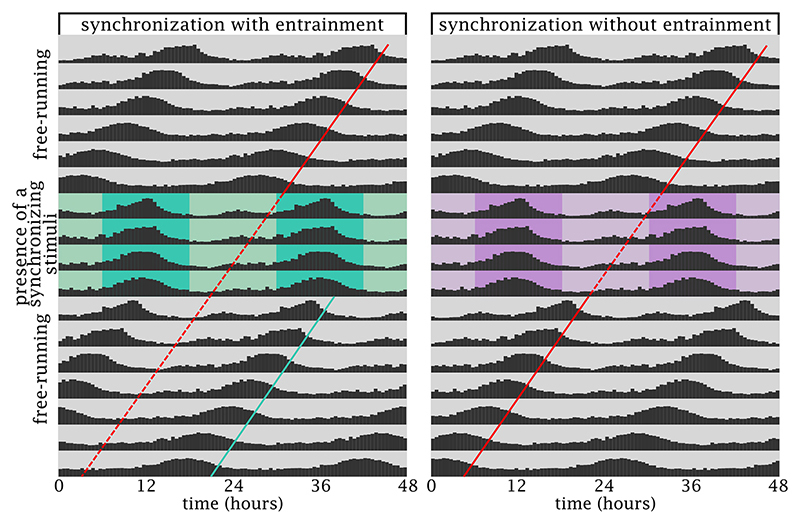
True-entrainment versus synchronization of activities without a proper entrainment of the circadian clock. The two hypothetical actograms describe two fundamentally different situations. In both situations, a generic animal starts free-running without any environmental cues (grey background) and shows a slightly short period with a certain phase (red line). Then, each animal is confronted with an oscillating external stimulus (green or purple). In both cases, these stimuli are able to synchronize the activity to a 24h cycle. The key difference is that the green stimulus is of such nature that it manages to entrain the clock, and after the removal of the stimulus, the activity follows from the new phase (green line), free-running with the same periodicity but in a different phase. In the case of the purple stimulus, the behaviour goes back to the original red phase, ignoring the experience of the purple stimulus. The plots represent fabricated double-plotted actograms, where the y-axis indicates the days of the experiment, each row represents two consecutive days, and the black bars indicate the magnitude of a certain behaviour or physiological property that is oscillating in a circadian fashion with one peak every circa 24h. The x-axis represents time in hours, when external cues are present the time is commonly referred to as Zeitgeber Time (ZT), and the standard is to indicate ZT0 as the initiation of the stimulus (typically light ON, the beginning of the day). When the animals is free running (no external cues), time is referred as Circadian Time (CT).

For all living organisms, the presence or absence of conspecifics and other members of their ecosystem are critical for survival. Therefore, synchronizing their activities in relation to the potential for interactions, whether inter- or trans-specific, is equally crucial. There is abundant evidence of synchronization phenomena, whether to coexist in time and space or to avoid such interactions. However, distinguishing between synchronization of events and an entrainment of the circadian system that establishes a long lasting phase over time, allowing anticipation of an up-coming change in conditions, is not an easy task regardless of the nature of the synchronizing stimulus. Figure 1 shows two different theoretical examples where an animal that is free running with its own circadian time (CT), established by the endogenous period and phase of its internal clock, is subjected to a training event. During this event, the animal shows synchronization to the oscillation in the stimulus, and it runs with the *zeitgeber* time (ZT). Only when the presence of the external stimulus ends one can evaluate if the *zeitgeber* was able to impact the master clock and set a new phase (Figure 1, left) or the animals returns to its original phase (Figure 1, right). Thus, evidence that synchronization is based on a true entrainment of the circadian clock, i.e., a modification of the phase of these internal rhythms, requires an experimental approach that it is not always present or accessible.

From an experimental perspective, it requires precise and challenging interventions that are often very difficult to perform. The primary challenge lays on the direct influence of social inputs on behaviour, which can alter the expression of daily cycles without necessarily impacting the intrinsic circadian clock or can activate temporal conditioning mechanisms. When a stimulus induces behaviours at a time away from their typical circadian phase, it is described as “masking” the genuine circadian phase. This veiling effect makes it laborious to discern whether observed behavioural changes are a result of circadian clock entrainment or merely a response to immediate social cues. Researchers face the complexity of distinguishing between genuine circadian modulation and behavioural responses induced by external stimuli, requiring meticulous and original experimental designs to tease apart these entangled interactions (Aschoff, 1960; Mrosovsky, 1999).

The circadian timekeeping system in humans, as in other mammals, is governed by environmental cues such as light quality and intensity, and temperature (Dibner et al., 2010). This system is synchronized to geophysical time predominantly through photic cues detected by the retina. Short exposures to low light intensity efficiently phase-shift the circadian clock, suppresses melatonin production, and induces alertness (Chang et al., 2012). In line with that, individuals with total blindness often exhibit circadian rhythm abnormalities, with a significant proportion of subjects experiencing free-running rhythms and some struggling with recurrent insomnia and daytime sleepiness (Sack et al., 1992). This underscores the pivotal role of the light-dark cycle in synchronizing the human circadian system. However, groundbreaking experiments done by one of the founding fathers of modern chronobiology, Jürgen Aschoff, in 1971 highlighted the profound influence of social cues as primary entrainment factors in humans (Aschoff *et al*., 1971). Carrying out experiments in total darkness, he and his team showed that social cues are key to entrain human circadian rhythms. They showed that in the absence of light but within a strong social context, the expected drift in physiological or psychological functions was not observed, underscoring the potency of social cues in orchestrating circadian regulation. Then, the growing idea that emerges is that maintaining alignment between photic and non-photic cues is paramount for the proper functioning of the circadian clock. Disruptions can manifest in various forms, including a mismatch between an individual’s self-selected cycles of light and dark and the natural day-night cycle. Several socially imposed factors, such as shift work, daylight saving time, and geographic location within a time zone, as well as travelling across longitudes, have been explored as potential sources of circadian disruption. These factors serve as particularly relevant situations for studying the consequences of circadian misalignment on health, shedding light on the intricate relationship between environmental cues, circadian rhythms, and their implications for human well-being. 

**Figure 2 -:**
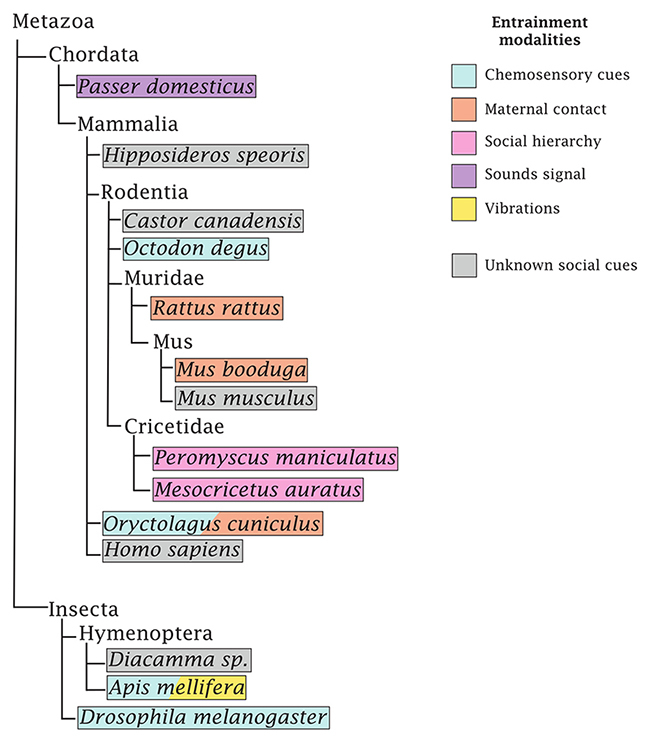
Sensory modalities of the social cues that entrain the circadian clock across species. A schematic representation of the taxonomic relationship (based on the NCBI Taxonomy) between animals mentioned along the review. The different colours represent the diverse range of modalities that have been tested to have an influence on the circadian clock.

Beyond the reported effect of social entrainment in humans, many extraordinary examples came from a variety of species in which we have different degrees of knowledge about the role of socialization on the clock and the mechanistic underpinnings (Figure 2). Experiments on bats (*Hipposideros speoris*) that inhabit caves inherently provide a context of socialization in constant darkness, serving as a valuable biological model for investigating non-photic entrainment of circadian rhythms (Marimuthu et al., 1978). Seminal work revealed that caged bats, when surrounded by free-flying conspecifics, exhibit synchronization of activity with colony-wide patterns (Marimuthu *et al*., 1981). This observation points to the ability of bats to communicate and socially synchronize their circadian rhythms. Furthermore, the study found that isolated bats placed in a cave of a different species free run in their circadian activity, indicating a species-specific element in the synchronization process. It is worth mentioning that this initial line of experiments did not explore the mechanisms of entrainment, the potential of these cues to induce phase shifts or the effect they have on the molecular clock. However, they do unravel the social dynamics of bats in their natural habitats and contribute to our understanding of species-specific influences on circadian regulation in the absence of external environmental light. Another similar example of social synchronization in nature comes from a study on the Canadian beaver (*Castor canadensis*) during winter, period in which they remain in their lodges covered of snow and ice, deprived of light inputs (Bovet and Oertli, 1974). A compelling series of recordings showed that a family of animals free-run with a single coherent 27-hour rhythm, rather than isolated individual rhythms, supporting the idea that phase synchrony was sustained by social interactions.

There are also well documented examples of the lack of effect of social interaction on circadian activity rhythms in certain conditions. For instance, experiments with the nocturnal sugar glider (*Petaurus breviceps*), a small omnivorous Australian possum, showed that members of a pair maintained under constant illumination are not mutually synchronized and free-run with different circadian period lengths. While this particular set of experiments did not show social synchronization, it does not exclude the possibility of this phenomenon in this species. A similar experiment but studying deer mice (*Peromyscus maniculatus*) showed proper social synchronization in which the activity rhythm of the dominant mouse entrains the activity rhythm of the subordinate (Crowley and Bovet, 1980). On the same line, solid evidence showed entrainment and phase shifting to brief agonistic encounters between male golden hamsters (*Mesocricetus auratus*) (Mrosovsky, 1988). These experiments showed that repeated social interactions at the same time of day could entrain activity rhythms in a way consistent with the shape of the phase response curves, placing social cues on an equal footing with light as a *zeitgeber*. As well as same-sex interaction, opposite-sex interactions are key for fitness. Work with mice (*Mus musculus*) has shown that both male and female free-running subjects exposed to the opposite sex exhibited a phase advance at circadian time 3 (CT3), delay at CT21, and no response at CT9, while only males responded with a phase advance at CT15 (Sonker and Singaravel, 2021). Beyond the clear effect of social activity on the circadian clock, these experiments also point out to a gender difference in circadian clock responses for social cues, drawing a more complex and interesting landscape. Finally, probably the most elementary social interaction is the bond between a caring progenitor and its offspring. Evidence from different mammals demonstrated that maternal contact is crucial for coordinating the circadian clock during late fetal and early neonatal life, and this interaction is a well-documented example of effective social entrainment of the circadian clock (Sumova et al., 2006). In rats (*Rattus rattus*), it has been shown that maternal care improves offspring’s circadian system and health (Olejnikova et al., 2018). Moreover, the circadian rest-activity cycles of pups in the night-active mouse *Mus booduga* are regulated by cyclic presence and absence of their mother, playing a role as *zeitgeber* (Viswanathan and Chandrashekaran, 1985). This is also the case for new-born rabbit (*Oryctolagus cuniculus*) pups, which suprachiasmatic nuclei (SCN), the master pacemaker in the mammalian brain, show endogenous 24‐h rhythmicity in the expression of the clock genes Per1, Per2, and Bmal1, and it is reported that these rhythms are entrained by a one-a-day nursing event (Caldelas et al., 2009). 

## The mechanisms behind social entrainment

As mentioned, while there is ample evidence of synchronization by social cues and good evidence for true entrainment of the molecular clock, understanding the underlying mechanisms of circadian entrainment in response to social cues poses challenges in experimental design and execution. Mechanistic data supports the idea that light is the main entrainment cue for most animals, meanwhile, social *zeitgebers* could reach circadian clocks through different pathways. For instance, social cues could represent and induce a variety of internal states and employ pathways encoding stress, arousal, and fear or directly elicit locomotion. Alternatively, social information can reach the master clock via sensory-specific input pathways that transduce visual, tactile, auditory, gustatory and/or olfactory information.

Behavioural arousal in mammals can induce non-photic circadian clock resetting and modulate its response to light-dark cycles, potentially influencing human circadian rhythms (Webb et al., 2014) (Mistlberger and Antle, 2011). This non-photic entrainment has both biological and therapeutic implications, and finds applications in experimental design for behavioural neuroscience studies. Importantly, arousal-induced phase shifts in the circadian clock are mediated by the cholinergic forebrain arousal system that reaches the SCN (Yamakawa et al., 2016). This is evidenced by atropine (a potent competitive and reversible antagonist of the muscarinic acetylcholine receptors) injections into the SCN that block arousal-induced phase shifts. These observations highlight the pivotal role of cholinergic pathways in the complex interplay between behavioural arousal and circadian rhythms. In addition, the thalamic intergeniculate leaflet plays a described role in non-photic entrainment of the circadian clock of Syrian hamsters (*Mesocricetus auratus*), with its loss affecting non-photic entrainment and activity onset (Maywood et al., 1997).

## The mechanisms behind social entrainment: the case for sound social cues

Avian circadian rhythms, akin to those observed in mammals, regulate a spectrum of vital biological processes such as sleep-wake cycles, visual function, song production, migratory patterns, and orientation. The parallels between avian and mammalian circadian systems position birds as valuable models for understanding the dynamics of human biological clocks. Studies involving house sparrows (*Passer domesticus*) have presented compelling evidence that sound cues possess the capacity to shape circadian rhythms (Menaker and Eskin, 1966), representing a pivotal stride in our comprehension of the entrainment process. These findings mark the inaugural demonstration of a direct correlation between sound stimuli and the modulation of a biological clock, reminiscent of the role of vibrations in honeybees (Beer et al., 2016) (see section below). Moreover, one notable experiment elucidated that the introduction of a musically enriched bird song stimulus during the early subjective day phase can induce a significant advancement in circadian rhythms in humans (Goel, 2006). This advancement is postulated to stem from an arousal-mediated effect previously described, underscoring the multifaceted nature of the mechanisms involved. Intriguingly, the non-photic stimulus not only influences the timing of the circadian rhythm but can also dictate whether the resynchronization leans towards a phase advance or delay.

The intricate interplay between auditory cues and the temporal alignment of biological clocks, as well as the underlying mechanisms governing it, awaits further elucidation. Importantly, investigations with animals more amenable to genetic manipulations may prove instrumental in unravelling the intricacies of these mechanisms, as well as other perceptual modalities. A key study employing the fruit fly *Drosophila melanogaster* shed light on the role of diurnal variations in mechanical vibrations as potential resetters of circadian clock phases (Simoni et al., 2014). Although the source of this mechanical vibration in nature can be of many sorts, and not necessarily represent socially relevant information, the mechanistic insight allows further evaluation. The chordotonal organs, situated in limb joints of insects, provide the animals with neural impulses for posture and position detection as well as sensitivity to mechanical stimuli, specifically vibrations and movements in their environment, reminiscent of the mammalian ear. This organ plays a role in coordinating how temperature affects circadian cycles in flies (Sehadova et al., 2009) and has the potential of acting as mediator for the feedback of various behaviours onto the endogenous biological clock. In this regard, the processing of mechanosensory inputs to entrain the circadian clock of *Drosophila melanogaster* is well described (Simoni *et al*., 2014). Specifically, the daily locomotor activity of wild-type flies can be synchronized using cycles of 12 hours of vibration and 12 hours of quiet. Behavioural synchronization to sound cycles requires a functional clock and chordotonal organs, and are accompanied by phase alterations in the daily oscillations in the abundance of the central clock protein PERIOD in the brain pacemaker. This highlights the crucial role of proprioceptive organs in providing feedback for maintaining the alignment of circadian rhythm in correspondence to stimulus-induced activity. As with sound-induced entrainment in birds and humans, the molecular intricacies of how mechanical vibrations integrate into the circadian system remain an intriguing area for future exploration, where studies with genetically amenable organisms may prove fundamental.

## The mechanisms behind social entrainment: the case for olfactorysocial cues

Odorants have a key evolutionary significance for various fitness-related behaviours such as mate choice, predator avoidance, and foraging strategies. Many studies suggest that social olfactory cues contribute to circadian entrainment and the resetting of the clock in various species, influencing physiological, behavioural rhythms, and memory recall.

**Figure 3 -:**
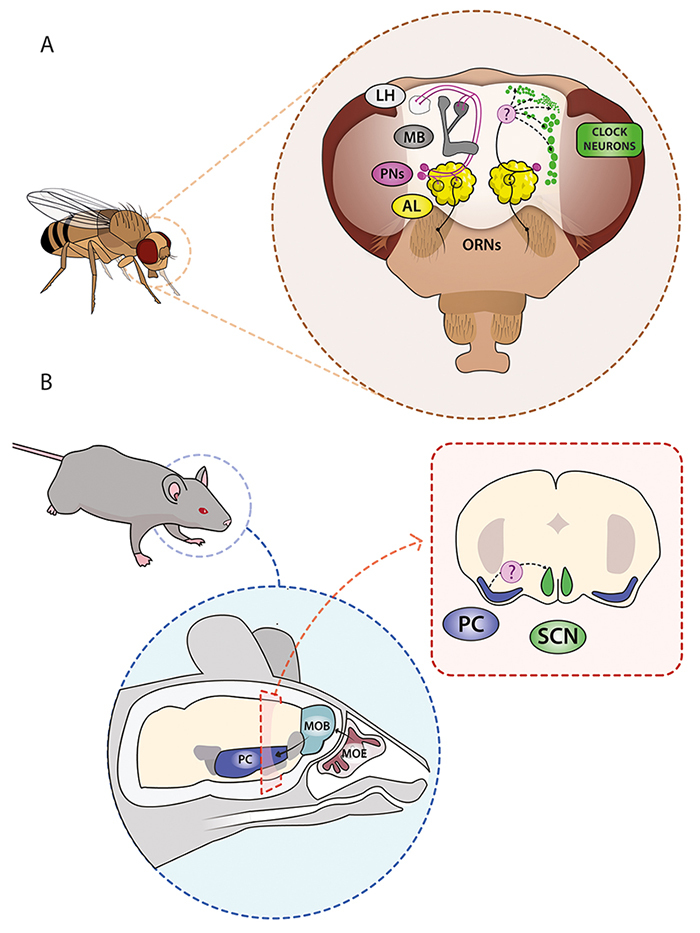
Olfactory cues are key to social entrainment in both mammals and insects. A. For the genetic model system *Drosophila melanogaster*, there is a very precise description of the detection and processing of chemosensory cues, as well as a detailed mapping of the clock neurons and their connectivity. In particular, olfactory molecules bind to olfactory receptor neurons (ORNs) located at the antennae and maxillary palps. These cells send their projections to the antennal lobe (AL) where they form glomeruli and make synapses with projection neurons (PNs), the output neurons of the AL. These cells send projections to the dorsal protocerebrum, transmitting the olfactory information to multimodal processing structures, such as mushroom bodies (MBs) and lateral horn (LH). These projection neurons are the main candidates to drive relevant social information to the clock cells, either by direct synaptic contact or by means of intermediate neuronal circuits that con involve neuromodulators or neuropeptides, both common tools of the circadian system. Thus, we still do not know how the olfactory system and the circadian network are connected (dashed arrows marked with a question mark in the scheme). B. In mammals, we also have a precise description of both, the olfactory system and the circadian pacemaker, the suprachiasmatic nucleus (SCN). In particular, the vertebrate olfactory system is analogous to the insect system: the olfactory receptor neurons are located on the olfactory epithelium and send their axons to the olfactory bulb (OB), where they make synapses with mitral cells that project to other areas of the brain, such as the piriform cortex (PC). It is postulated that this area is critical to send social olfactory cues relevant to the SCN, but the connection between the olfactory system and the circadian network is still not described.

Odorants can act as a circadian time cue, influencing circadian locomotor behaviour in mammals (Figure 3), with effects even more pronounced in the absence of the SCN, but require the presence of a functional canonical molecular clock (Abraham et al., 2013). In term of mechanisms, the entrainment of rabbit pups to maternal nursing described earlier provides a well-established example (Caldelas et al., 2009). Maternal olfactory cues in the milk, particularly the mammary pheromone 2MB2, effectively synchronize the circadian system of new-born rabbits, affecting core body temperature, locomotor activity, and metabolic variables (Caldelas *et al*., 2009; Montufar-Chaveznava et al., 2013). At the same time, the relevance of odorants is not always sufficient for entrainment. One study in mice reported proper entrainment upon social interaction, while exclusive odorant stimulation was not enough to entrain the clock and only produced masking of the overt activity rhythms (Sonker et al., 2021). Importantly, in a different set of pairing experiment, it has been shown that social interactions with conspecifics of the opposite-sex up-regulated c-fos/C-FOS in the olfactory bulb, per-1/PER-1 in the SCN, C-FOS, and PER-1 in the piriform cortex (a brain region involved in the processing and perception of olfactory information) of both male and female when the interaction between a free-running individual and an entrained one is presented at CT3; pointing to a clear involvement of key genes in olfactory and circadian dedicated areas (Sonker and Singaravel, 2021). 

A highly social and diurnal rodent indigenous to central Chile offered fundamental insights into the entrainment of circadian clocks by social olfactory cues. The common degus (*Octodon degus*) is known for its communal burrows and cooperative behaviours such as communal nesting and nursing each other’s young, providing an ecological context where social cues may be particularly relevant. A series of works suggests that the circadian clock of this rodent is not solely influenced by external stimuli like light but also responds to social information (Hagenauer and Lee, 2008). Importantly, the observed entrainment-dependent mechanisms and variations in chronotype parallel those found in humans. In particular, pairing experiments conducted with animals in a free-running condition unveiled that social information, in the absence of light, holds the capacity for partial entrainment. Such social cues induce changes in period and phase of free-running rhythms. Interestingly, sex-specific differences were noted in this entrainment capacity (Goel and Lee, 1996). Free-running females housed with entrained females exhibited faster entrainment compared to those housed with entrained males. Moreover, in this context, an increased frequency of scent-marking behaviours was observed in comparison to same-sex housing conditions. Crucially, the same group of researchers underscored the relevance of the olfactory system in the entrainment process (Governale and Lee, 2001). Olfactory bulbectomy experiments conclusively demonstrated that the olfactory bulbs and chemosensory cues play a necessary role in socially facilitated entrainment (Goel and Lee, 1997). This finding provides robust evidence that chemosensory stimuli can modulate the circadian system in a diurnal rodent, shedding light on the intricate interplay between social cues, olfaction, and circadian regulation; however, the mechanistic connection between the olfactory and circadian systems is not described. In agreement with this line of research, experiments employing isolated honeybees also point towards the importance of olfactory cues involved in circadian entrainment within the colony (see section below).

In this regard, key mechanistic insight comes from the social circadian entrainment in the fruit fly *Drosophila melanogaster* (Figure 3). Experiments show that group-housed flies compared to isolated flies exhibit better phase coherence, meaning that the circadian clocks of the flies housed together are more synchronous than those of isolated flies (Levine et al., 2002). Furthermore, social interactions between genetically arrhythmic and wild type flies generate a more dispersed peak of locomotor activity, consequently, worsening the phase coherence in the rhythmic group. This shows that the genetically encoded circadian clock drives the expression of synchronizing olfactory cues that influence the overt circadian rhythm of surrounding conspecific. Moreover, the size of the group of flies is important on the effect of the social context in the circadian clock since larger groups induce a more prominent effect and pairwise social interactions do not show alterations (Lone and Sharma, 2011). Critically, it has been shown that odorants may be responsible for the social synchronization of the circadian rhythm described before. Wild type flies who received “fly air” (a group of individuals who received pumped air from a vial with food and flies) show a stronger synchronization than flies who received “neutral air” (air from a vial without flies) (Levine *et al*., 2002). This means that individuals who only received chemosensory signals from another group of flies synchronize their phase of circadian activity. Continuing with this line of experiments, social interactions between olfactory-impaired mutants and genetically arrhythmic flies did not alter the circadian rhythm of the first group, different from what was shown before with the effect of the arrhythmic flies on the wild type ones (Levine *et al*., 2002). This result supports the idea that without a functional olfactory sensory system, the circadian clock of flies cannot be entrained by a social context under constant darkness. However, the olfactory impaired mutant employed is a pleiotropic mutation in a voltage-gated sodium channel, and a more specific olfactory mutant needs to be tested to fully support the conclusion.

In order to dissect the chemosensory signalling that entrains the circadian clock of flies under social interactions, Krupp et al. (2008) performed various experiments analysing the pheromone expression only in male flies and how this was correlated with the circadian rhythm at the molecular level. They demonstrated that three key clock genes (*period*, *timeless* and *Clock*) are expressed in oenocytes, the epidermal cells responsible for the production of cuticular hydrocarbons in insects, implying that these cells could act as peripheral clock. Some of these hydrocarbons can act as pheromones, and their accumulation on the body surface is *per*-dependent. Social interactions between heterogeneous groups (wild-type and arrhythmic male flies) and homogeneous groups (genotypically identical wild-type individuals) showed different patterns of clock gene expression in oenocytes. Furthermore, these two treatments differentially affect the overall levels of several cuticular pheromones and their temporal patterns. This evidence supports the hypothesis that pheromones are responsible for the synchronization of the phase of circadian activity in male flies.

Although the nature of the signal and the system that detects it seems clear, the neuronal mechanisms that convey the signals to the circadian clock have not been elucidated. A key piece of evidence shows that electroantennogram responses to an appetitive olfactory stimulus show circadian variability in their amplitude (Krishnan et al., 1999). A molecular clock that depends on the genes *period* and *timeless* controls this rhythm, but it is independent of the pacemaker neurons that mediate locomotor activity rhythms. This finding supports the hypothesis of the presence of peripheral oscillators in the antennae that mediate a circadian sensitivity to odorants (Krishnan *et al*., 1999; Tanoue et al., 2004; Krishnan *et al*., 2008). 

Altogether, these experiments propose the interesting hypothesis that circadian entrainment by social cues may rely on both the cyclic production of odorants and an oscillation in olfactory perception, demonstrating the complexity of the process and the degree of evolutionary pressure to remain in synchrony with the environment. 

## Time synchronization in eusocial insects

Regarding the role of social cues to organize physiology and behaviour, true social, or eusocial, animals are probably the epitome in the animal kingdom, and the role of these cues on the circadian clock has been studied too. Eusociality refers to an advanced level of social organization in which individuals within a group display reproductive division of labour, cooperative care of offspring and overlapping generations. 

In particular, ants represent an extremely diverse group of animals showing eusocial organizations with highly developed social communication skills that modulate their behaviour. The knowledge of the molecular clock in ants has grown exponentially, however our understanding of it is limited when compared with other model systems, such as flies or bees. Specifically, as other animals, ants also show 24 hours oscillations on the expression pattern of the central clock gene *period* (Ingram et al., 2009; Das and de Bekker, 2022). An interesting study by Fujioka et al. (2017) on the role of social cues on the circadian rhythms of nurse ants (*Diacamma sp.*) showed that isolated nurses show circadian rhythms in locomotion. However, nurses that were kept with conspecifics in the first stages of development, such as eggs and larvae (that require high levels of care by the nurse) show weaker oscillation, with around the clock activity levels, suggesting that the task performed by the animals (or cues associated to the task) are relevant information that can modulate the overt locomotor rhythms. 

To study if the role of an ant worker in a group can influence the temporal pattern of activity, Fujioka et al. (2019) tracked the rest-activity profile of individual ants that were housed in groups that included animals with different roles (i.e. young nurses and old foragers) and found that these interactions induced the loss of the rhythmic activity. In isolation, both young and old worker ants showed circadian activity rhythms that were abolished when these workers were grouped with other workers from different age groups (Fujioka *et al*., 2019).

In a follow up study, the authors tracked large groups of *Diacamma sp.* (between 88 to 194 animals) and also found that workers of different ages (young and old) did not show circadian oscillation in their locomotion when grouped (Fujioka et al., 2021). These results suggest that the socialization provided by the colony has a clear effect on the locomotor pattern of activity of individual ants. Nevertheless, the nature and identity of the social cues underlying this influence over the locomotion rhythms have not been identified yet. In conclusion, these data show that the breeding needs of the colony can impose an effect on the activity of individual nurses and control their behaviour regardless of the entrained circadian rhythms. What remains to be addressed is the effect of nursing behaviours on the endogenous circadian rhythms of individual ants, distinguishing between effects on the clock entrainment or a masking effect on the locomotion levels. At the same, in future experiments would be critical for the understanding of the ant’s circadian system to focus on a different output, considering that the locomotor activity may be detached from a circadian control, but many aspects of the animal physiology and behaviour may remain running under the control of the circadian clock, and demonstrate daily oscillations.

Another fascinating example of true social organization are honeybees (*Apis mellifera*). They exhibit a highly organized colony structure where the cooperative efforts of the different castes contribute to the overall success of the colony. Within a hive, there is a single reproductive female, the queen, with the primary role of laying eggs. The rest of the female animals, the worker bees, assume various tasks such as foraging, nursing and defending the colony depending on their age. Bees exhibit very developed communication skills that includes pheromones, food exchange, temperature signals and even complex behaviours such as dances, further exemplifying the importance of collaboration for the thriving of the colony (Seeley, 1995). Are social cues capable of entraining honeybees’ circadian clock? In an environment as complex as a hive it is very challenging to identify the most important *zeitgebers*. For instance, light is mostly not accessible for the many workers that remain inside the hive. Also, given the fact that temperature is such an important factor for breeding it is tightly regulated. Based on laboratory experiments that suggest that at least 6° to 10° C degrees oscillations are necessary to entrain bees (Moore and Rankin, 1993; Fuchikawa and Shimizu, 2007), the temperature inside the hive is not the most likely factor to serve as a *zeitgeber*. However, early studies in honeybees in which the investigators entrained bees to different phases and then put them together suggested that bees can socially synchronize to a common activity phase (Moritz and Kryger, 1994). The authors showed that two groups of bees that were separated by a partition that allowed airflow improved their synchronization to a common daily rhythm. These early results provided a lot of evidence to the hypothesis that volatiles are crucial for social synchronization. Given the fact that the animals were able to touch each other, the role of direct contact was not eliminated. However, by using coloured food, the authors were able to prove that trophallactic (mouth-to-mouth) contacts were not involved in social synchronization given more support to the volatile hypothesis.

In addition to volatiles and trophallaxis, another important socialization mechanism is the direct contact between animals. Nevertheless, experiments performed by different groups suggest that direct contact is not needed for bees to experience social synchronization of activity (Beer et al., 2016; Fuchikawa et al., 2016). In particular, Fuchikawa *et al.* (2016) performed their experiments with honeybees within the hive with the goal of uncoupling masking from entrainment. These experiments were the first to clearly show that social cues can be as powerful, or even more, than photic cues to entrain the circadian clock of an insect. They show that the social cues of the hive can entrain the clock in animals that are experiencing conflicting information. Exposing newly emerged workers to the colony environment for two days was sufficient to show strong entrainment to the colony phase. The synchronization experienced by the bees in the hive, compared to individually isolated sisters could come from direct interactions with other animals or by a synchronizing effect of the colony environment. To test this, the authors set animals to interact outside the hive or placed the bees in a cage inside the hive precluding physical contact but allowing access to volatile as well as mechanical information. The results of these experiments showed that interaction with conspecifics is important, since the phase coherence of the group of animals that interacted outside the hive improved, but, more importantly, the hive environment is *per se* capable of synchronizing animals that were isolated inside the hive (Fuchikawa *et al*., 2016). 

On the other hand, Beer and colleagues utilized a novel and insightful experimental set up that allows monitoring the activity rhythms of individual honeybees while they are exposed to the social context of a colony (Beer *et al*., 2016). To do this, they placed bees in individually monitored tubes that allowed them to be in contact with environmental cues from the hive. By doing so, they were able to record the activity of individual animals while they are exposed to social cues from the hive. In one experiment the animals were exposed to conflicting *zeitgeber* cues, i.e. an outdoor colony was transfer to a shifted light-dark (LD) cycle in an incubator. Isolated animals synchronized to the LD cycle faster, whereas animals that were exposed to the signals coming from the hive kept their phase in synchrony and took significantly longer to re-entrain to the new LD set by the incubator light schedule. Additionally, they wanted to evaluate the role of direct contact on social synchronization, and by using an experimental device that separated the animals from the colony by a double mesh, they showed that direct contact is not necessary for honeybees to experience social synchronization (Beer *et al*., 2016). It has also been proposed that other activities performed by the workers could be the source of the social entrainment, such as airborne and substrate-borne vibrations, volatile pheromones, hive odours, or gases such as CO_2_ or O_2_ (Moore and Rankin, 1993; Fuchikawa and Shimizu, 2007; Ohashi et al., 2009). In particular, studying the relevance of substrate-born vibration it has been shown that bees that were individually housed in chambers within the same tray have higher coupling strength values compared to bees at a similar distance but caged on a different, mechanically isolated, tray. These results clearly show that vibrations can play a role in social synchronization. Additionally, individually caged animals placed in one tray with interconnecting tubes that allows the transfer of volatiles, and also allows the animals to touch and lick each other, had a stronger phase coherence compared to bees on the same tray but housed in unconnected cages. These results also point out the relevance of olfactory cues involved in this process; however, these experiments are not sufficient to rule out the role of direct contact on social synchronization. 

A key set of experiment that proves the true entrainment nature of social synchronization in honeybees comes for the work of Siehler and Bloch (2020). They showed that bees exposed for six days to the volatiles of a foraging hive show a closer phase relationship with foraging bees of the same hive compared with bees exposed to an empty hive of a comparable size. The key contribution is that the rhythmic activity was measured after removing the animals from the environment that was used to entrain them. This shows that properties of the internal clock have been modified, rather than attributing the changes solely to the masking effect of social cues.

In conclusion, social entrainment can be a potent and stable *zeitgeber*, and even overriding photic entrainment of circadian rhythms in honeybees, challenging again the view that light plays the dominant role in animal circadian entrainment. However, in many contexts we still do not understand if the phase of the circadian clock is truly altered, or whether synchrony and segregation of activity respond to a masking effect, or a learning process based on a circadian time sense. In addition, the question of which are the volatiles that are the synchronizing cues remains. Some authors propose that volatile molecules such as pheromones or fluctuations in the concentration of gases, such as CO_2_ or O_2_, that depend on the level of activity of the animals could be the actual signals that convey the social synchronization, but the actual relevant signal(s) remain elusive.

## Concluding remarks

Investigating the circadian entrainment by social cues opens avenues for exploring the intricate relationship between an organism’s internal state and its external environment. Unravelling the molecular and neural pathways involved in this process will enhance our comprehension of how animals adapt to their surroundings and optimize their chances of survival. 

Additionally, understanding the influence of social information on the circadian clock has implications across various disciplines, including neuroscience, psychology, and chronobiology. Internal misalignment with the external cues due to travelling across time-zones (jetlag), as well as social jetlag, the disruptions in circadian rhythms due to socially imposed schedules, has been associated with adverse health outcomes, including sleep, psychiatric and metabolic disorders. By investigating how social information can entrain the circadian clock, we can develop strategies to mitigate the negative effects of jetlag and optimize the synchronization of our internal clock. These lines of research will foster interdisciplinary discussions and inspire future collaborations aimed at enhancing our understanding of human and animal health, contributing to a more holistic perspective on well-being.
